# The Associations of Serum IL-37 With the Severity and Prognosis in Patients With Community-Acquired Pneumonia: A Retrospective Cohort Study

**DOI:** 10.3389/fimmu.2021.636896

**Published:** 2021-05-07

**Authors:** Jia-Le Wang, Xue Chen, Yi Xu, Yue-Xin Chen, Jing Wang, Yu-Lu Liu, Hai-Tao Song, Jun Fei, Hui Zhao, Lin Fu

**Affiliations:** ^1^ Second Clinical Medical College, Anhui Medical University, Hefei, China; ^2^ Respiratory and Critical Care Medicine, Second Affiliated Hospital of Anhui Medical University, Hefei, China; ^3^ Department of Toxicology, Anhui Medical University, Hefei, China

**Keywords:** community-acquired pneumonia, IL-37, inflammatory cytokine, community-acquired pneumonia severity score, biomarker

## Abstract

**Background:**

Recent evidences suggested that IL-37 may participate in the pathophysiology of community-acquired pneumonia (CAP). Nevertheless, its exact biological role was unknown. The objective of this study was to determine the associations of serum IL-37 with the severity and prognosis in CAP patients based on a retrospective cohort study.

**Methods:**

The whole of 120 healthy subjects and 240 CAP patients were summoned. Peripheral blood was collected and IL-37 was detected using ELISA.

**Results:**

Serum IL-37 was obviously decreased in CAP patients on admission. In addition, serum IL-37 was gradually decreased in parallel with CAP severity scores. Correlative analysis revealed that serum IL-37 was negatively associated with CAP severity scores and inflammatory cytokines. Further logistical regression found that reduction of serum IL-37 augmented the severity of CAP patients. Moreover, the follow-up research was performed in CAP patients. Serum lower IL-37 on admission prolonged the hospital stay in CAP patients. Serum IL-37 combination with PSI and CURB-65 had a stronger predictive capacity for death than IL-37 and CAP severity score alone in CAP patients.

**Conclusion:**

There are remarkably negative correlations between serum IL-37 with the severity and prognosis in CAP patients. Serum IL-37 on admission prolongs the hospital stay, demonstrating that IL-37 may involve in the process of CAP. Serum IL-37 may be regarded as a biomarker for diagnosis and prognosis for CAP patients.

## Introduction

Community-acquired pneumonia (CAP) is a common illness and main cause of morbidity and mortality all over the world, particularly in adults over 60 years old and in children. Despite the quick development of new drugs and therapy, CAP still evokes high risks of complications and death (1–3). Previous studies reported that 120 million cases (14 million severe cases) with pneumonia were diagnosed in children below 5 years old in 2010. The incidence of CAP needing hospitalization is 20.6 cases per 10,000 every year (4, 5). Pneumonia has contributed to more than 60000 deaths, 1.2 million hospitalizations, 2.3 million emergency department visits and $10 billion in hospital costs in the United States annually (6, 7). It elevates economic burden and medical resources for individuals and society at large. Because several obvious clinical symptoms are low predictive capacity sometimes, the most challenging task for a physician is to decrease the mortality and severity for CAP patients. Timely and accurate diagnosis and evaluation as well as useful biomarkers are essential for decreasing the mortality for CAP patients. Although CAP severity scores have been extensively applied in the diagnose, the existence of inconsistency in the different scoring systems and many indices are used comprehensively, which elevates the use difficulty for now available scoring systems. Therefore, an effective biomarker is urgently needed to decrease mortality, elevate survival rate and prolong life span for CAP patients.

Interleukin-37 (IL-37) is a relatively new discovered member of the IL-1 family, formerly termed IL-1 family member 7 (IL-1F7) (8). Recent milestone studies demonstrated that IL-37 exerts important roles in limiting innate inflammation, suppressing acquired immunity as well as significantly downregulating several inflammatory cytokines induced by IL-1 and Toll-like Receptors (TLR) (9, 10). Previous studies have found that transgene expression of human IL-37 obviously repressed inflammatory reaction in mice models of endotoxic shock, colitis, myocardial infarction, lung and spinal cord injury (8, 11–13). Moreover, pretreatment with recombinant human IL-37 inhibited *Aspergillus* infection-induced NLRP3 inflammasome activation, IL-1β secretion, and lung injury (14). In vivo animal study found IL-37 was decreased in the mice model with idiopathic pulmonary fibrosis (IPF) and IL-37 repressed TGF-β signaling in IPF fibroblasts (15). In addition, an experimental study in vivo found that IL-37 transgenic mice evoked excessive inflammation and tissue damage in murine *pneumococcal pneumonia* (16). Moreover, mounting evidences revealed that IL-37 plays a crucial protective role in non-small cell lung cancer, murine allergic asthma, fibrosis, and airway inflammation by alleviating inflammation (17–19). These results indicated that IL-37 may involve in the pathophysiological process of several pulmonary diseases.

However, the role of IL-37 in CAP was obscure now. The associations of IL-37 with the severity and prognosis in CAP patients were unknown. We speculate that IL-37 exert an important function in the pathogenesis of CAP. Nevertheless, the clinical and experimental evidences about the physiological function of IL-37 in CAP were absence. Consequently, the goal of this study was to explore the role of IL-37 and analyze the associations of serum IL-37 with the severity and prognosis in CAP patients based on a retrospective cohort study.

## Methods

### Study Design and Subjects

**Figure 1:**
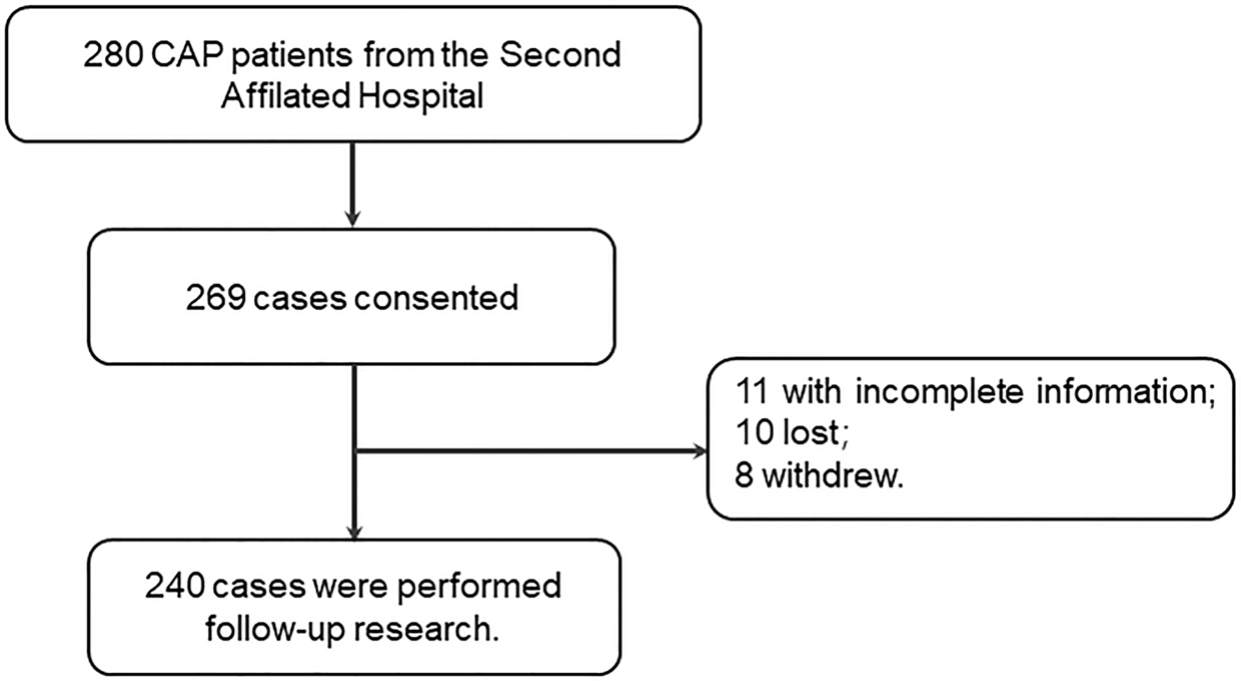
Flow diagram of recruitment and follow-up study in CAP patients.

A flowchart describing the research design is provided in **Figure 1**. Two hundred and eighty patients were recruited and agreed to take participate in the follow-up research. Eleven patients with incomplete information, 10 lost cases and eight withdrawing cases were ruled out in this research. At last, 240 CAP patients taken participate in the present research. This study was approved by Ethics Committee of Anhui Medical University and met the principles stated in the Declaration of Helsinki. All patients or patients’ next of kin provided written and informed consent.

Patients with CAP admitted to the Department of Respiratory and Critical Care Medicine in Second Affiliated Hospital of Anhui Medical University, from May 2018 to June 2020. The research group included all CAP patients who had no an attack or underwent hospitalization in the last six months. All CAP patients who were enrolled in this research met the diagnosis criteria of CAP (20). The exclusion criteria included: severely immunocompromised; pregnant women; patients accompanied with pulmonary malignancies and active tuberculosis; hospital stay less than 5 days. Demographic characteristics and clinical information were gathered from electronic medical record system. The No-CAP group consisted of healthy subjects from the physical examination center. The inclusion criteria for the control group were healthy status and normal pulmonary function. The No-CAP cases were not permitted to have any of the (chronic) pulmonary or non-pulmonary diseases that could affect the results in this research. Serum samples were collected from all CAP patients before any therapeutic or drug intervention were conducted. Complete blood routine examination was conducted in the clinical laboratory of Second Affiliated Hospital. The severity of CAP patients was evaluated using CAP severity scores, mainly included Pneumonia Severity Index (PSI), CURB-65 (confusion, urea nitrogen, respiratory rate, blood pressure and age less than 65 years), CRB-65 (confusion, respiratory rate, blood pressure and age less than 65 years), SMART-COP (systolic blood pressure, multilobar chest radiograph, albumin respiratory rate, tachycardia, confusion, oxygenation and arterial pH) and CURXO (confusion, urea, respiratory rate, x‐ray, oxygen, and over 80 years) (21–23).

### Enzyme-Linked Immunosorbent Assay (ELISA)

Fasting blood samples (5 ml) were collected. Blood samples were centrifuged and immediately stored in the −80°C super cold refrigerator based on our previous studies (24, 25). ELISA kits of inflammatory cytokines (TNF-α, JYM0110Hu; IL-6, JYM1942Hu; IL-1β, JYM0083Hu) were purchased from Wuhan ColorfulGene Biological Technology Co. IL-37 ELISA kits (CSB-E16185h) were purchased from Cusabio, Wuhan, China (https://www.cusabio.com/). Serum inflammatory cytokines were detected strictly according to the manufacturer’s instructions (26).

### Statistical Analysis

All analyses were carried out using GraphPad Prism 5.0 and SPSS 19.0 software. Demographic characteristics and clinical information were compared using Student’s *t* tests, Chi-square tests or Manne-Whitney U tests between two groups. Continuous variables were exhibited with median and interquartile range (IQR). Correlational analyses were conducted with linear regression and logistical regression. A *P*-value of ≤0.05 was considered statistically significant.

## Results

### Patient Demographics

In total, 240 CAP patients and 120 healthy subjects were enrolled in the present research. Demographic characteristics and clinical information were collected and analyzed in **Table 1**. No difference of gender, age, BMI, systolic pressure and diastolic pressure was observed between CAP patients and No-CAP cases. Moreover, blood routine examination indicated that the counts of white blood cells (WBCs), neutrophils, the ratios of platelet-lymphocyte (PLR), monocyte-lymphocyte (MON) and neutrophil-lymphocyte (NLR) were increased, the count of lymphocytes was decreased in CAP patients (**Table 1**). Meanwhile, serum parameters of liver function and renal function were detected. There was no obvious difference of ALT, AST, urea nitrogen, and creatinine between two groups. Only uric acid was slightly decreased in CAP patients (**Table 1**). Furthermore, several inflammatory cytokines were measured. The results suggested that the levels of inflammatory cytokines (TNF-α, IL-6, CRP, and IL-1β) were significantly elevated in CAP patients (**Table 1**). In addition, in-hospital mortality and 30-day mortality were 9.17% and 6.67% in CAP patients, respectively.

**Table 1:** Demographic and biochemical characteristics between CAP patients and control subjects.

Variables	CAP (n=240)	Control (n=120)	P
Male, n (%)	137 (57.2)	65 (54.0)	0.125
Age (years)	67.0 (52.0, 78.0)	64.0 (49.0, 81.0)	0.356
BMI	22.7 (20.1, 26.2)	21.8 (19.0, 25.2)	0.254
Systolic pressure (mmHg)	123.5 (108.0, 136.0)	124.5 (104.5, 144.5)	0.562
Diastolic pressure (mmHg)	74.0 (66.3, 82.8)	72.5 (65.5, 85.0)	0.335
WBC (10^9^/L)	6.93 (5.00, 9.55)	5.66 (4.55, 6.75)	<0.05
Neutrophil (10^9^/L)	4.88 (3.03, 7.21)	3.11 (2.44, 3.92)	<0.01
Lymphocyte (10^9^/L)	1.25 (0.84, 1.87)	2.28 (1.91, 2.54)	<0.01
NLR	3.89 (2.09, 9.08)	1.49 (1.31, 2.29)	<0.05
MON	0.33 (0.21, 0.57)	0.18 (0.16, 0.23)	<0.05
PLR	184.6 (120.1, 344.5)	109.3 (88.5, 135.6)	<0.01
ALT (U/L)	21.2 (13.1, 40.6)	19.3 (11.2, 38.9)	0.658
AST (U/L)	24.6 (20.1, 38.6)	23.6 (16.5, 41.2)	0.748
Urea nitrogen (mmol/L)	5.36 (4.12, 7.22)	4.68 (3.58, 5.32)	0.068
Creatinine (μmol/L)	60.2 (49.1, 77.8)	63.5 (48.5, 80.3)	0.298
Uric acid (μmol/L)	282.5 (200.3, 334.3)	395.3 (291.3, 460.2)	<0.05
TNF-α (pg/mL)	545.0 (282.8, 1200.8)	60.3 (35.6, 115.8)	<0.01
IL-6 (pg/mL)	78.6 (40.3, 101.3)	42.5 (18.6, 55.9)	<0.01
CRP (mg/L)	48.6 (3.8, 92.3)	11.3 (3.6, 25.6)	<0.01
IL-1β (pg/mL)	345.8 (182.3, 602.2)	63.5 (22.9, 98.6)	<0.01
In-hospital mortality, n (%)	22 (9.17%)	N.A.	N.A.
30-day mortality, n (%)	16 (6.67%)	N.A.	N.A.

WBC, white blood cell; PLR, platelet-to-lymphocyte ratio; MON, monocyte-to-lymphocyte ration; NLR, neutrophil-to lymphocyte ratio; ALT, alanine aminotransferase; AST, aspartate aminotransferase; N.A., Not available.

### The Levels of Serum IL-37 in Control Subjects and CAP Patients

**Figure 2:**
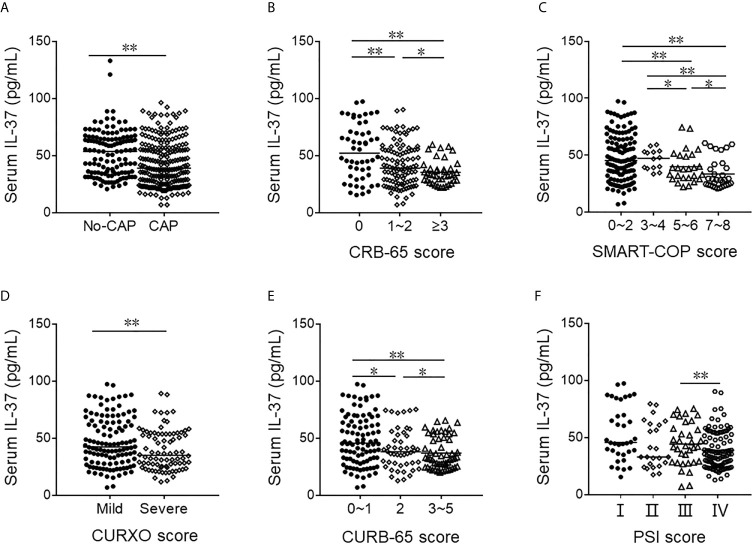
The levels of serum IL-37 in No-CAP and CAP cases. **(A–F)** The level of serum IL-37 was detected with ELISA. **(A)** The level of serum IL-37 in CAP patients and control subjects. **(B)** The level of serum IL-37 in different ranks of CRB-65 score of CAP. **(C)** The level of serum IL-37 in different ranks of SMART-COP score of CAP. **(D)** The level of serum IL-37 in different ranks of CURXO score of CAP. **(E)** The level of serum IL-37 in different ranks of SMART-COP score of CAP. **(F)** The level of serum IL-37 in different ranks of PSI score of CAP. *P < 0.05, **P < 0.01.

Serum IL-37 was detected between CAP patients and healthy subjects. As shown in **Figure 2A**, serum IL-37 was significantly decreased in CAP patients compared with control subjects. The severity of pneumonia was assessed with CAP severity scores, such as CURXO, PSI, CURB-65, CRB-65, and SMART-COP. In addition, serum IL-37 was compared among different grades of CAP patients. As shown in **Figure 2B**, serum IL-37 was gradually decreased in parallel with CRB-65 score. On the basis of SMART-COP score, we found that serum IL-37 was higher in the grades of 0 to 2 and 3 to 5 scores than those in the grades of 5 to 6 and 7 to 8 scores (**Figure 2C**). Moreover, serum IL-37 was further analyzed in CAP patients based on CURXO score. The results indicated that serum IL-37 was decreased in severe patients with CAP (**Figure 2D**). At the same time, we found that serum IL-37 was gradually reduced parallelly with CURB-65 score (**Figure 2E**). Based on PSI score, serum IL-37 was lower in the grade of IV than those in the grade of III (**Figure 2F**).

### Associations of Serum IL-37 With Different Parameters Among CAP Patients

The associations between serum IL-37 and CAP severity scores were assessed among CAP patients using linear regression. The results indicated that there were negative associations between serum IL-37 and CAP severity scores (**Table 2**). Moreover, associations between serum IL-37 and CAP severity scores were further analyzed using univariate and multivariate logistic regression. As shown in **Table 3**, the univariate logistic regression indicated that serum IL-37 was negatively associated with PSI (β=0.892; 95% CI: 0.786, 0.991), SMART-COP (β=0.865; 95% CI: 0.711, 0.965), and CURXO (β=0.861; 95% CI: 0.765, 0.948). Furthermore, the associations between blood routine parameters and serum IL-37 were analyzed in all CAP patients. These results suggested that serum IL-37 was negatively and significantly associated with PLR (**Table 2**). In addition, there was no associations of serum IL-37 with the parameters of liver function and renal function (**Table 2**). Besides, serum IL-37 was negatively associated with TNF-α and IL-1β in CAP patients (**Table 2**).

**Table 2:** Correlations between serum IL-37 and different parameters in CAP patients.

Variables	CURB-65	CRB-65	PSI	CURXO	SMART-COP
***r***	-0.216	-0.218	-0.216	-0.272	-0.247
***P***	0.032	0.030	0.028	<0.001	0.014
Variables	WBC	Neutrophil	Lymphocyte	NLR	MON
***r***	-0.027	0.126	0.116	-0.139	-0.045
***P***	0.793	0.215	0.254	0.172	0.659
Variables	PLR	Uric acid	Urea nitrogen	Creatinine	ALT
***r***	-0.240	0.050	-0.100	-0.152	0.019
***P***	0.017	0.625	0.329	0.136	0.849
Variables	AST	TNFα	IL-1β	CRP	IL-6
***r***	-0.123	-0.193	-0.163	-0.112	-0.084
***P***	0.225	0.010	0.030	0.333	0.553

**Table 3:** Associations between serum IL-37 and CAP severity scores among CAP patients.

Variables	Univariabte (95% CI)	P	Multivariabte (95% CI)*	P
CURB-65	1.016 (0.992, 1.040)	0.194	0.990 (0.964, 1.016)	0.435
CRB-65	0.998 (0.890 1.254)	0.675	1.002 (0.974, 1.030)	0.913
PSI	0.892 (0.786, 0.991)	0.002	0.901 (0.735, 0.986)	0.004
SMART-COP	0.865 (0.711, 0.965)	0.033	0.911 (0.854, 0.985)	0.044
CURXO	0.861 (0.765, 0.948)	0.023	0.886 (0.756, 0.968)	0.040

*Adjusted for age and sex.

### The Association Between Serum IL-37 and the Prognosis in CAP Patients

**Figure 3:**
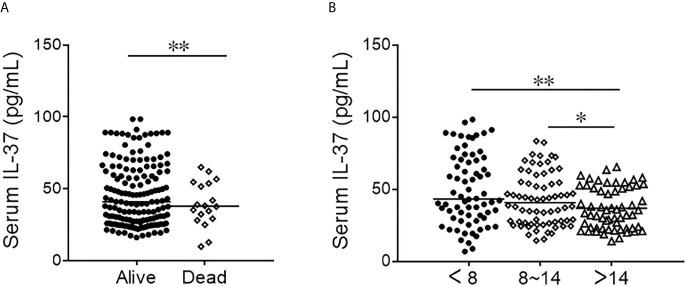
The levels of serum IL-37 in alive and dead CAP patients. **(A, B)** The level of serum IL-37 was detected with ELISA. **(A)** The level of serum IL-37 in alive and dead CAP patients. **(B)** The level of serum IL-37 in different hospital stay of CAP patients. *P < 0.05, **P < 0.01.

Serum IL-37 was compared between alive and dead patients on admission. As shown in **Figure 3A**, serum IL-37 was decreased in dead cases. Moreover, we found that serum IL-37 in CAP patients with more than 14 days hospital stay was lower than in the hospital stay of less than 14 days (**Figure 3B**). In addition, the association between serum IL-37 and the prognosis of CAP patients was analyzed using logistical regression. The univariate logistic regression revealed that serum IL-37 was inversely associated with hospital stay (β=0.901; 95% CI: 0.815, 0.976) (**Table 4**). In order to eliminate confounding factors, the multivariate logistic regression was continued to conducting. We found that serum IL-37 was negatively associated with the hospital stay (β=0.886; 95% CI: 0.716, 0.965). However, there was no association of serum IL-37 on admission with the risk of death in CAP patients (**Table 4**).

**Table 4:** Association between serum IL-37 and the prognosis among CAP patients.

Variables	Univariate (95% CI)	*P*	Multivariate (95% CI)*	*P*
Hospital stay	0.901 (0.815, 0.976)	0.015	0.886 (0.716, 0.965)	0.018
Death	1.020 (0.984, 1.057)	0.274	0.983 (0.946, 1.020)	0.360

*Adjusted for age and sex.

### ROC Curves and Cutoff Point Analysis for Serum IL-37

**Figure 4:**
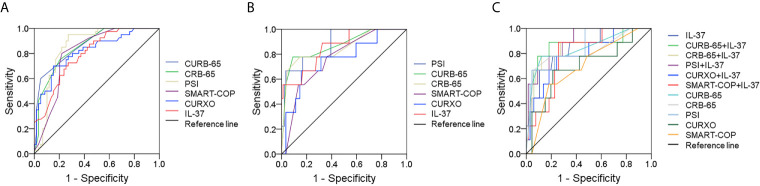
Receiver operating characteristic curves for different predictive biomarkers on admission. **(A)** ROC curve was used to evaluate the severity of different predictive biomarkers for CAP. **(B)** ROC curve was used to evaluate the death risk of different predictive biomarkers for CAP. **(C)** ROC curve was used to evaluate the death risk of serum IL-37 combination with CAP severity scores.

The severity of predictive capacity between different CAP severity scores and serum IL-37 was conducted with receiver operating characteristic (ROC) area under the curve (AUC). As shown in **Figure 4A**, the AUCs of severity were as follows: IL-37, 0.819 (95% CI: 0.695, 0.896); CURB-65, 0.835 (95% CI: 0.765, 0.921); CRB-65, 0.865 (95% CI: 0.798, 0.912); PSI, 0.865 (95% CI: 0.769, 0.915); SMART-COP, 0.866 (95% CI: 0.765, 0.965); CURXO, 0.865 (95% CI: 0.763, 0.922). Besides, the optimal cutoff value of IL-37 for CAP severity was 41.36 pg/ml, with a specificity of 76% and sensitivity of 85% (**Figure 4A**). Moreover, the death of predictive capacity between different CAP severity scores and serum IL-37 was performed *via* ROC area analysis. As shown in **Figure 4B**, the AUCs were as follows: IL-37, 0.855 (95% CI: 0.729, 0.982); CURB-65, 0.757 (95% CI: 0.585, 0.928); CRB-65, 0.850 (95% CI: 0.698, 1.001); PSI, 0.883 (95% CI: 0.774, 0.991); SMART-COP, 0.766 (95% CI: 0.625, 0.909); CURXO, 0.702 (95% CI: 0.551, 0.856). Besides, the optimal cutoff value of IL-37 for the death was 20.11 pg/ml, with a specificity of 79% and sensitivity of 81% (**Figure 4B**). Finally, in order to further use serum IL-37 as a biomarker in the diagnosis for CAP patients, serum IL-37 was cutoff in the level of 20.11 pg/ml. The predictive power of death of IL-37 combined with CAP severity scores was analyzed. As shown in **Figure 4C**, though there was no difference of AUC between IL-37 combination with SMART-COP, CRB-65, and CURXO with single IL-37 or CAP severity score, the AUCs were larger in PSI+IL-37 (0.911, 95% CI: 0.782, 0.986), CURB-65+IL-37 (0.898, 95% CI: 0.704, 0.981) than IL-37 and single CAP severity score alone in CAP patients.

## Discussion

This was the first clinical study to investigate the associations of serum IL-37 with the severity and prognosis in patients with CAP based on a retrospective cohort study. The findings of this research mainly include: (1) serum IL-37 on admission was decreased in CAP patients; (2) serum IL-37 on admission was gradually decreased in parallel with the severity in CAP patients; (3) serum IL-37 on admission was negatively associated with the severity in CAP patients; (4) serum lower IL-37 on admission was positively associated with the hospital stay of CAP patients; (5) serum IL-37 combination with PSI and CURB-65 had a stronger predictive capacity for death than IL-37 and CAP severity score alone in CAP patients.

The previous studies found that IL-37 played significant roles in repressing innate inflammation, acquired immunity and inflammatory cytokines (9, 10). Transgene human IL-37 obviously repressed inflammatory reaction in many inflammatory diseases (8, 11–13). Besides, IL-37 was decreased in the model of idiopathic pulmonary fibrosis (15). In addition, IL-37 overexpression caused excessive inflammatory reaction in the models of *streptococcus pneumoniae* (16). However, there is no previous study on the role of IL-37 in CAP patients. Therefore, we detected the level of serum IL-37 between CAP patients and control cases. In the present research, we found that the level of serum IL-37 was decreased in CAP patients compared with healthy subjects. Moreover, we found that serum IL-37 was gradually decreased in parallel with CAP severity scores. Correlation analysis found that there was a negative correlation between serum IL-37 and the severity in CAP patients. In addition, logistical regression analysis indicated that serum lower IL-37 elevated CAP severity scores. These results demonstrate that serum IL-37 is negatively associated with the severity of CAP patients.

More and more data suggested that inflammation involved in the pathogenesis of CAP, promoted the occurrence and development of CAP (27, 28). Several inflammatory cytokines were increased and inflammatory signaling pathways were activated in the progress of CAP (17, 29). The levels of pro-inflammatory cytokines were compared between two groups in our research. We found that pro-inflammatory cytokines were increased in CAP patients. IL-37 was inversely associated with inflammatory cytokines. Moreover, it is generally believed that blood routine parameters were changed in the infected patients and have been proposed as indicators of systemic inflammation and infection (30). We found that the count of lymphocytes was decreased in CAP patients. The numbers of WBCs and neutrophils, PLR, MON and NLR were increased in CAP patients. However, we did not observe the associations between serum IL-37 and blood routine parameters in CAP patients. Similarly, there was no association of serum IL-37 with liver function and renal function. Besides, the association between serum IL-37 and the prognosis of CAP has been explored. Logistical regression analysis indicated the level of serum IL-37 on admission was reduced in CAP patients with longer hospital stay. Not only that, the predictive capacities of serum IL-37 and CAP severity scores were compared through ROC curve test. Though there was no obvious difference of severity prediction capacity between serum IL-37 and CAP severity scores, the predictive power of death is higher in serum IL-37 than those in CURB-65, SMART-COP, and CURXO. Meanwhile, CAP patients whose serum IL-37 level is less than 20.11 pg/ml were needed to pay more attention. It is essential to take effective measures to reduce the mortality of CAP patients for clinicians. Antibiotics treatment, respiratory support, circulation support, electrolyte balance and acid-base equilibrium are adopted in the severe CAP patients. Further analysis found that serum IL-37 combination with PSI and CURB-65 had a stronger predictive capacity for death than single IL-37 and CAP severity score in CAP patients. These results demonstrate serum IL-37 may serve as a potential diagnostic and prognosis biomarker for CAP.

The function of IL-37 in CAP patients is scarcely clear. The growing data showed that IL-37 not only served as a natural suppressor of innate inflammatory and immune responses but also repressed antigen-specific adaptive immunity (9, 31). In addition, some researches indicated over-expression of IL-37 significantly inhibited inflammation in the models of multiple organ injury (8, 11–13). Meanwhile, IL-37 protected organ injury through alleviating inflammatory reaction (32–34). Collectively, these data suggested that IL-37 is regarded as a new anti-inflammatory cytokine. Now, *Streptococcus pneumoniae*, as well as infections with other pathogens, induce an inflammatory response and CAP. IL-37 can exert anti-inflammatory roles in the process of CAP. So, the reduction of IL-37 may aggravate inflammatory reaction and the severity of CAP patients.

There are a few limitations in the present research. Firstly, this was a retrospective cohort study based on hospital-population, a prospective cohort study is needed in the next work. Secondly, this was only a correlation analysis, the causation of serum IL-37 reduction and CAP remained unknown. Further animal experiments *in vivo* would help resolve this confusion in the future. Thirdly, this was a single-center and small sample size study, a larger sample size and multicenter study are required. Fourthly, IL-37 was only measured in the serum, the levels of IL-37 in sputum and bronchoalveolar lavage fluid are unknown in CAP patients.

## Conclusions

In summary, the present study mainly analyzed the correlations of serum IL-37 with the severity and prognosis in CAP patients through a retrospective cohort study. We found that serum IL-37 on admission is decreased in CAP patients. The level of serum IL-37 is gradually decreased parallelly with the severity of CAP patients. Lower serum IL-37 on admission increases the risk of longer hospital stay in CAP patients. Serum IL-37 alone or in conjunction with CAP severity scores have a better predictive capacity for death in CAP patients. These results imply that IL-37 may involve in the pathophysiology of CAP. Therefore, it is reasonable to presume that serum IL-37 may be regarded as a biomarker for diagnosis and prognosis in CAP patients.

## Data Availability Statement

The raw data supporting the conclusions of this article will be made available by the authors, without undue reservation.

## Ethics Statement

Written informed consent was obtained from the individual(s) for the publication of any potentially identifiable images or data included in this article.

## Author Contributions

J-LW, XC, YX, Y-XC, JW, Y-LL, H-TS, and JF performed the research. LF and HZ designed the research study. LF analyzed the data and wrote the manuscript. LF provided funding support. All authors contributed to the article and approved the submitted version.

## Funding

This study was supported by National Natural Science Foundation Incubation Program of the Second Affiliated Hospital of Anhui Medical University (grant number: 2020GQFY05) and the Scientific Research of Health Commission in Anhui Province (grant number: AHWJ2021b091).

## Conflict of Interest

The authors declare that the research was conducted in the absence of any commercial or financial relationships that could be construed as a potential conflict of interest.
